# Evodiamine Mitigates Cellular Growth and Promotes Apoptosis by Targeting the c-Met Pathway in Prostate Cancer Cells

**DOI:** 10.3390/molecules25061320

**Published:** 2020-03-13

**Authors:** Sun Tae Hwang, Jae-Young Um, Arunachalam Chinnathambi, Sulaiman Ali Alharbi, Acharan S. Narula, Ojas A. Namjoshi, Bruce E. Blough, Kwang Seok Ahn

**Affiliations:** 1 Department of Science in Korean Medicine, Kyung Hee University, 24 Kyungheedae-ro, Dongdaemun-gu, Seoul 02447, Korea; suntaeh12@gmail.com (S.T.H.); jyum@khu.ac.kr (J.-Y.U.); 2 Department of Botany and Microbiology, College of Science, King Saud University, Riyadh -11451, Saudi Arabia; carunachalam@ksu.edu.sa (A.C.); sharbi@ksu.edu.sa (S.A.A.); 3 Narula Research, Chapel Hill, NC 27516, USA; anarula1@nc.rr.com; 4 Center for Drug Discovery, RTI International, Research Triangle Park, Durham, NC 27709, USA; onamjoshi@rti.org (O.A.N.); beb@rti.org (B.E.B.)

**Keywords:** evodiamine, c-Met, STAT3, prostate cancer, apoptosis

## Abstract

Evodiamine (EVO) is an indoloquinazoline alkaloid that exerts its various anti-oncogenic actions by blocking phosphatidylinositol-3-kinase/protein kinase B (PI3K/Akt), mitogen-activated protein kinase (MAPK), c-Met, and nuclear factor kappa B (NF-κB) signaling pathways, thus leading to apoptosis of tumor cells. We investigated the ability of EVO to affect hepatocyte growth factor (HGF)-induced c-Met/Src/STAT3 activation cascades in castration-resistant prostate cancer (CRPC). First, we noted that EVO showed cytotoxicity and anti-proliferation activities in PC-3 and DU145 cells. Next, we found that EVO markedly inhibited HGF-induced c-Met/Src/STAT3 phosphorylation and impaired the nuclear translocation of STAT3 protein. Then, we noted that EVO arrested the cell cycle, caused apoptosis, and downregulated the expression of various carcinogenic markers such as B-cell lymphoma 2 (Bcl-2), B-cell lymphoma-extra large (Bcl-xL), cyclin D1, cyclooxygenase 2 (COX-2), survivin, vascular endothelial growth factor (VEGF), and matrix metallopeptidases 9 (MMP-9). Moreover, it was observed that in cPC-3 and DU145 cells transfected with c-Met small interfering RNA (siRNA), Src/STAT3 activation was also mitigated and led to a decrease in EVO-induced apoptotic cell death. According to our results, EVO can abrogate the activation of the c-Met/Src/STAT3 signaling axis and thus plays a role as a robust suppressor of tumor cell survival, proliferation, and angiogenesis.

## 1. Introduction

Prostate cancer remains a major cause of mortality annually among males [1,2,3,4,5,6]. In 2015, prostate cancer was the fifth commonly diagnosed cancer in South Korea and it is expected to become the fourth in 2019 [7,8,9]. Moreover, prostate cancer is predicted to be the seventh cause of mortality in men in 2019 [9]. Therefore, the incidence of prostate cancer in South Korea is rapidly increasing [8,10]. When prostate cancer is diagnosed, the tumor can be treated by surgery, radiotherapy, chemotherapy, and hormonal therapy (androgen deprivation) [11,12]. Androgen deprivation therapy (ADT) remains the commonly prescribed treatment for prostate cancer patients [13,14]. Unfortunately, this therapy is not curative and leads to the development of metastatic androgen-independent prostate carcinoma that is significantly resistant to existing therapeutic interventions [15]. Thus, there exist an unmet need to identify treatment options for castration-resistant prostate cancer (CRPC).

c-Met is a receptor expressed in epithelial cells that can be induced by hepatocyte growth factor (HGF) [16]. Activated c-Met can trigger the phosphorylation of downstream mitogen-activated protein kinase (MAPK) and phosphatidylinositol-3-kinase (PI3K) signaling pathways that can mediate cellular growth, survival, and invasion [11,16]. Furthermore, Src tyrosine kinase has also been suggested as a downstream target molecule in the c-Met cascade [16]. In clinical studies, c-Met expression has been frequently observed in metastatic and CRPC, and higher level of HGF can be associated with poorer outcomes in prostate cancer patients [17,18,19].

A number of drugs isolated from nature have shown potential against different cancers including prostate [20,21,22,23,24,25,26,27,28,29,30,31,32]. Evodiamine (EVO) is an indoloquinazoline alkaloid reported to have various pharmacological effects including anti-proliferation [33,34,35], and anti-tumor properties [36,37], and can cause both cell cycle arrest [38,39] and apoptosis [40,41] in vitro and in vivo. According to previous studies, EVO can effectively block PI3K/protein kinase B (Akt), MAPK, and nuclear factor kappa B (NF-κB) signaling pathways and enhance apoptosis [33,42,43,44]. In this study, it was observed that EVO has an anti-proliferation effect in androgen-independent prostate cancer PC-3 and DU145 cells and can lead to apoptosis through attenuating c-Met/Src/STAT3 signaling pathways.

## 2. Materials and Methods

### 2.1. Reagents

**Figure 1:**
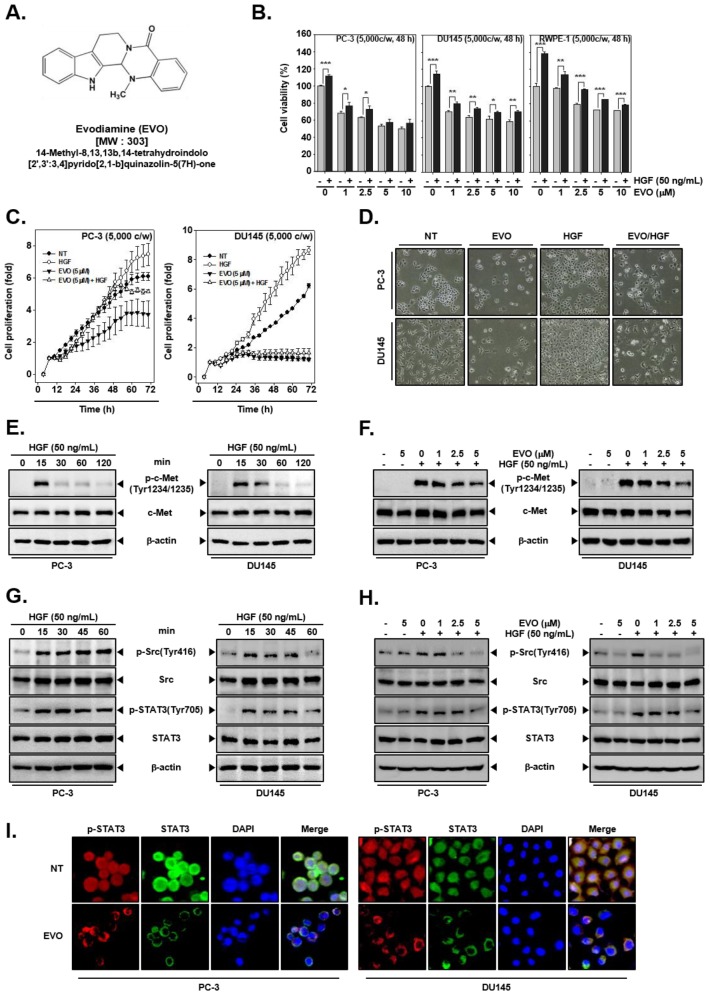
Inhibition of cell growth by evodiamine (EVO) and hepatocyte growth factor (HGF)-induced c-Met/Src/STAT3 phosphorylation in human prostate cancer cells. (**A**) Chemical structure of EVO. (**B**) PC-3 (5 × 10^3^ cells/well), DU145 (5 × 10^3^ cells/well), and normal human prostate (RWPE-1) (5 × 10^3^ cells/well) cells were pre-treated with EVO (0, 1, 2.5, 5, 10 µM) for 1 h and then treated with HGF (50 ng/mL) for a total of 48 h. Cell viability was analyzed by the 3-(4,5-dimethylthiazol-2-yl)-2,5-diphenyltetrazolium bromide (MTT) assay. (**C**) PC-3 (5 × 10^3^ cells/well) and DU145 cells (5 × 10^3^ cells/well) were treated with EVO or HGF. cell proliferation was determined by using real-time cell analysis (RTCA). (**D**) PC-3 (1 × 10^5^ cells/well) and DU145 cells (5 × 10^4^ cells/well) were treated with EVO or HGF. After 48 h of treatment, cell morphology and density were observed by an optical microscope at a 100x magnification. (**E**) PC-3 (5 × 10^5^ cells/well) and DU145 cells (5 × 10^5^ cells/well) were treated with HGF (50 ng/mL) for the indicated times to determine when c-Met activation occurred. Whole cell extracts were prepared and immunoblotted with antibodies for phospho-c-Met(Tyr1234/1235) (p-c-MET) and c-Met. (**F**) PC-3 (5 × 10^5^ cells/well) and DU145 cells (5 × 10^5^ cells/well) were pre-treated with EVO for 4 h and then treated with HGF for 15 min. Whole cell extracts were prepared and immunoblotted with antibodies for phospho-c-Met(Tyr1234/1235) and c-Met. (**G**) PC-3 (5 × 10^5^ cells/well) and DU145 cells (5 × 10^5^ cells/well) were seeded in six-well plates and incubated overnight in serum-free conditions. Then, they were treated with HGF (50ng/mL) in serum-free conditions for the indicated times to determine when Src and STAT3 were activated. Whole cell extracts were prepared and immunoblotted with antibodies for phospho-c-Src(Tyr416), Src, phospho-STAT3(Tyr705), and STAT3. (**H**) PC-3 (5 × 10^5^ cells/well) and DU145 cells (5 × 10^5^ cells/well) were seeded in six-well plates and incubated overnight in serum-free conditions. Then, they were pre-treated with EVO for 4 h and treated with HGF for 15 min in serum-free conditions. Whole cell extracts were prepared and immunoblotted with antibodies for phospho-c-Src(Tyr416), Src, phospho-STAT3(Tyr705), and STAT3. (**I**) PC-3 (3 × 10^4^ cells/well) and DU145 cells (3 × 10^4^ cells/well) were treated with 5 µM EVO for 4 h. The samples were subjected to immunocytochemistry with phospho-STAT3(Tyr705) and STAT3 antibodies. Non-treated (NT) and EVO (−)/HGF (−) indicate control samples treated with medium containing 0.1% DMSO.

EVO (Figure 1A) was received from RTI International (Research Triangle Park, North Carolina, USA). We dissolved 10 mg of EVO in 3.3 mL of dimethyl sulfoxide (DMSO) to make a 10 mM stock solution and then diluted it to 1 mM in DMSO for use in the experiments. DMSO, 3-(4,5-dimethylthiazol-2-yl)-2,5-diphenyltetrazolium bromide (MTT), propidium iodide (PI), Tris base, glycine, NaCl, sodium dodecylsulfate (SDS), and bovine serum albumin (BSA) were purchased from Sigma-Aldrich (St. Louis, MO, USA). The terminal transferase-mediated dUTP–fluorescein nick-end labeling (TUNEL) assay kit was from Roche Diagnostics GmbH (Mannheim, Germany). The enhanced chemiluminescence (ECL) kit was from DoGenBio (Seoul, Korea).

### 2.2. Cell Lines and Culture Conditions

Human prostate cancer (PC-3 and DU145) cells and normal human prostate (RWPE-1) cells were obtained from the American Type Culture Collection (Manassas, VA, USA). All cells were incubated in RPMI medium containing 10% inactivated fetal bovine serum (FBS) and 1% penicillin/streptomycin. The cells were incubated at 37 °C in 5% CO2 conditions.

### 2.3. MTT Assay

Cytotoxicity was analyzed with the MTT assay. PC-3, DU145, and RWPE-1 cells were seeded on 96-well plates. Cells were pre-treated with EVO (0, 1, 2.5, 5, and 10 µM) for 1 h and then treated with HGF (50 ng/mL) for a total of 48 h. Then, the cells were treated with 2 mg/mL of MTT solution (30 µL/well) for 2 h and then with MTT lysis buffer (100 µL/well) for overnight incubation at 37 °C. Finally, after lysis, MTT formazan was measured by VARIOSKAN LUX microplate reader (Thermo Fisher Scientific Inc, Waltham, MA, USA) at 570 nm. Cell viability was expressed as relative percentages and normalized in comparison with that of untreated controls [45].

### 2.4. Real-Time Cell Proliferation Analysis (RTCA)

PC-3 and DU145 cells were seeded onto 16-well E-plates, integrated with gold microelectrode arrays, and real-time cell analysis (RTCA) was carried out with the xCELLigence System (Roche, Mannheim, Germany). Background impedance was measured in 100 µL of cell culture medium per well. The final volume was adjusted to 200 µL cell culture medium, including 5 × 10^3^ cells/well. After a 20 h initial incubation on the E-plates, all cells were treated with 5 µM of EVO with or without 50 ng/mL of HGF. Non-treated samples were used as controls. The cell index was monitored for 72 h, with measurements every 15 min [46,47].

### 2.5. Cell Morphology

PC-3 (1 × 10^5^ cells/well) and DU145 cells (5 × 10^4^ cells/well) were seeded on 12-well plate. The cells were treated with EVO (5 µM) and HGF (50 ng/mL) or non-treated for 48 h. Then the cells were observed using a Nikon ECLIPSE Ts2 microscope (Tokyo, Japan).

### 2.6. Western blot Analysis

Cells treated with various concentrations of EVO for the indicated times with or without HGF were washed with 1 × PBS (phosphate-buffered saline) and lysed. Then, protein concentrations were determined by Bradford reagent (Bio-Rad, Hercules, CA, USA), and equal amounts of whole cell lysates were prepared. Proteins were resolved by sodium dodecyl–polyacrylamide gel electrophoresis (SDS-PAGE) and transferred on nitrocellulose membranes. The membranes were blocked with 3% or 5% skim milk in 1× TBST (1× tert-butyldimethylsilyl (TBS) with 0.1% Tween 20) for 2 h at room temperature, and proteins were probed with target specific primary antibodies overnight at 4 °C. The membranes were washed with 1× TBST and incubated with horse radish peroxidase (HRP) at room temperature for 1 h. Then, proteins were detected by ECL (EZ-Western Lumi Femto, DoGenBio, Seoul, Korea) [48].

### 2.7. Immunocytochemistry for STAT3 Localization

PC-3 and DU145 cells treated with EVO with or without HGF were fixed in 4% paraformaldehyde (PFA) in 1× PBS for 20 min, permeabilized with 0.2% Triton X-100 for 10 min, and blocked with 5% BSA in 1× PBS for 1 h. The cells were probed with anti-phospho-STAT3(Tyr705) and -STAT3 antibodies overnight at 4 °C. The next day, the samples were washed in 1× PBS and incubated with the secondary antibodies Alexa Fluor^®^ 488 donkey anti-mouse IgG (H + L) and Alexa Fluor^®^ 594 donkey anti-rabbit IgG (H + L), at room temperature for 1 h, then washed in 1× PBS, stained with 1 µg/mL 4′,6-diamidino-2-phenylindole (DAPI) for 3 min, and washed again. The samples were mounted using a fluorescent mounting medium (Golden Bridge International Labs, Mukilteo, WA, USA) and then analyzed by using an Olympus FluoView FV1000 confocal microscope (Tokyo, Japan) [49].

### 2.8. Cell Cycle Analysis

To confirm apoptosis, we performed cell cycle analysis. PC-3 (5 × 10^5^ cells/well) and DU145 cells (5 × 10^5^ cells/well) were treated with EVO with or without HGF for the indicated time and at the indicated concentrations. After treatment, the cells were harvested and washed with 1× PBS, fixed in 100% ethanol, and incubated for 30 min at 37 °C with 0.1% RNase A in 1× PBS. The cells were then washed, resuspended, and stained in 1× PBS containing 25 µg/mL of PI for 30 min at room temperature. Then, the cells were analyzed by a BD Accuri™ C6 Plus Flow Cytometer (BD Biosciences, Becton-Dickinson, Franklin Lakes, NJ, USA) using the BD Accuri C6 Plus software [50].

### 2.9. TUNEL Assay

Late apoptotic cell death was determined using a Roche Diagnosis TUNEL assay kit. PC-3 (5 × 10^5^ cells/well) and DU145 cells (5 × 10^5^ cells/well) were treated with EVO with or without HGF for the indicated times and at the indicated concentrations and then were fixed in 4% paraformaldehyde at room temperature for 30 min, washed with 1× PBS, and permeabilized with 0.2% Triton X-100 at room temperature for 15 min. The cells were then washed with 1× PBS and resuspended in TUNEL reaction mixture for 1 h at 37 °C in the dark. DNA damage in the stained cells was analyzed by a BD Accuri™ C6 Plus Flow Cytometer (BD Biosciences, Becton-Dickinson, Franklin Lakes, NJ, USA) using the BD Accuri C6 Plus software [48].

### 2.10. Transfection of siRNA

The Neon™ Transfection System (Invitrogen, Carlsbad, CA, USA) was used for PC-3 and DU145 cells transfection by electroporation, using 100 nM of c-Met small interfering RNA (siRNA) and scrambled siRNA as a control, aliquoted into sterile microcentrifuge tubes. A Neon Tip was inserted into the Neon Pipette, and the mixture was aspirated into the tip avoiding air bubbles. The Neon Pipette was then inserted into the Neon Tube containing 3 mL of Neon Electrolytic Buffer E in the Neon Pipette Station. PC-3 cells were pulsed three times with a voltage of 1450 V and a pulse width of 10 ms and incubate for 24 h. DU145 cells were pulsed two times with a voltage of 1260 V and a pulse width of 20 ms and incubate for 24 h.

### 2.11. Statistical Analysis

Statistical significance of the data was measured using the Student unpaired *t*-test. Significance was set at *p < 0.05, **p < 0.01, and ***p < 0.001.

## 3. Results

### 3.1. EVO Suppressed Cellular Growth and Cell Proliferation.

Cell viability of prostate cancer cells was determined after separate and combined treatment with EVO and HGF. As shown Figure 1B, cells co-treated with EVO and HGF displayed higher viability than cells treated only with EVO, and the highest cell viability was noted for the group treated with HGF alone. Additionally, cell viability was found to be higher in normal prostate RWPE-1 cells than in prostate cancer PC-3 and DU145 cells upon exposure to EVO. To examine cell proliferation, PC-3 and DU145 cells were treated with 5 µM of EVO with or without 50 ng/mL of HGF. Proliferation of cells co-treated with EVO and HGF was higher again than that of cells treated only with EVO or HGF (Figure 1C). Moreover, upon morphological examination, EVO-treated cells displayed a decrease in the number of cells and a round morphology, whereas HGF-treated cells showed an increase in the number of cells, and EVO/HGF-co-treated cells showed a slight recovery in the cell number with respect to cells treated with EVO alone (Figure 1D).

### 3.2. EVO Attenauted c-Met/Src/STAT3 Phosphorylation in PC-3 and DU145 Cells.

The cells were treated with HGF (50ng/mL) for the indicated times to determine the optimum when c-Met became activated (p-c-Met). We observed that p-c-MET was maximally activated at 15 min in both cell types (Figure 1E). Phosphorylation of c-Met was induced only in HGF-treated PC-3 and DU145 cells, whereas EVO substantially downregulated c-Met phosphorylation, without changing c-Met expression (Figure 1F). We also determined the time point at which Src (p-Src) and STAT3 (p-STAT3) were activated. We noted that these two proteins were also phosphorylated after 15 min of treatment in both the cells types (Figure 1G). EVO downregulated HGF-induced phosphorylation of Src and STAT3 without affecting Src and STAT3 expression (Figure 1H). We also studied whether EVO can inhibit STAT3 nuclear translocation by using immunocytochemistry. As shown on Figure 1I, EVO effectively downregulated the movement of STAT3 to the nucleus in PC-3 and DU145 cells.

### 3.3. EVO Caused DNA Damage and Induced Apoptotic Cell Death.

**Figure 2:**
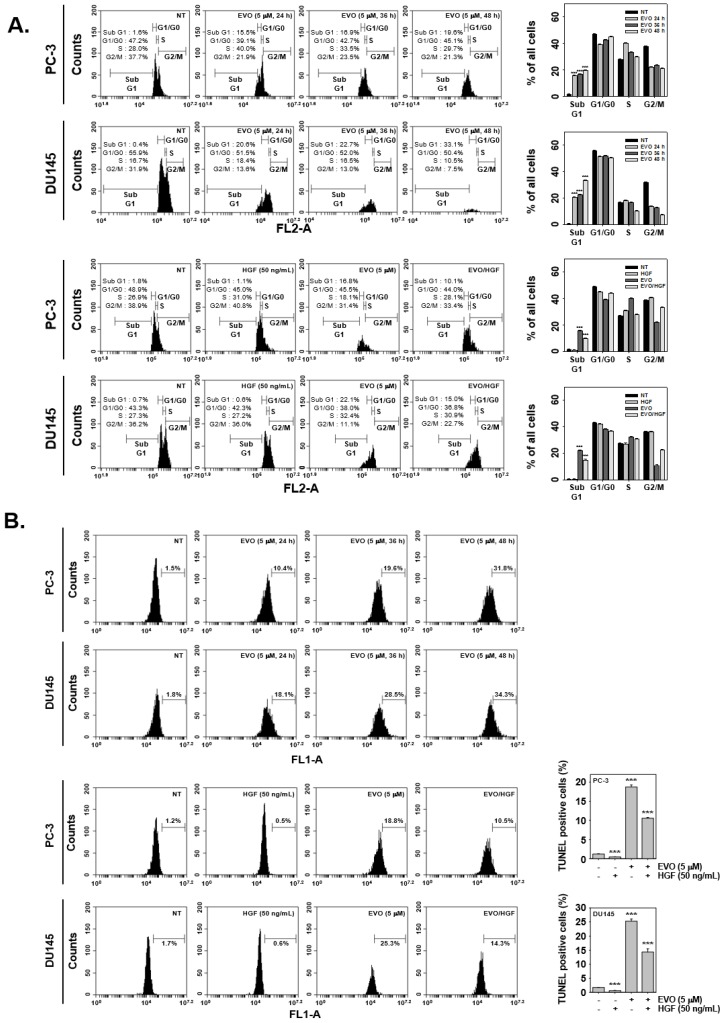
EVO arrests the cell cycle and induces apoptosis in PC-3 and DU145 cells. (**A**) PC-3 (5 × 10^3^ cells/well) and DU145 cells (5 × 10^3^ cells/well) were treated with EVO and HGF t the indicated concentrations and for the indicated times. The cells were digested with RNase A for 1 h, then stained with propidium iodide and subjected to flow cytometric analysis to monitor the cell cycle. (**B**) PC-3 (5 × 10^3^ cells/well) and DU145 cells (5 × 10^3^ cells/well) were treated with EVO or HGF at the indicated concentrations and for the indicated times. The cells were fixed, stained with TUNEL assay reagent, and then analyzed by flow cytometry. NT and EVO (−)/HGF (−) indicate control samples treated with medium containing 0.1% DMSO.

Cell cycle analysis and TUNEL assays were conducted to study apoptotic cell death by flow cytometry. The cell cycle results showed that EVO induced DNA damage in a time-dependent fashion. In cells co-treated with EVO and HGF, a decrease in DNA damage was observed with respect to cells treated with EVO alone (Figure 2A). Moreover, by employing the TUNEL assay it was noted that EVO induced apoptotic cell death in a concentration dependent fashion, but interestingly, in cells co-treated with EVO and HGF, apoptotic cell death was mitigated (Figure 2B).

### 3.4. EVO Downregulated the Expression of Carcinogenic Proteins.

**Figure 3:**
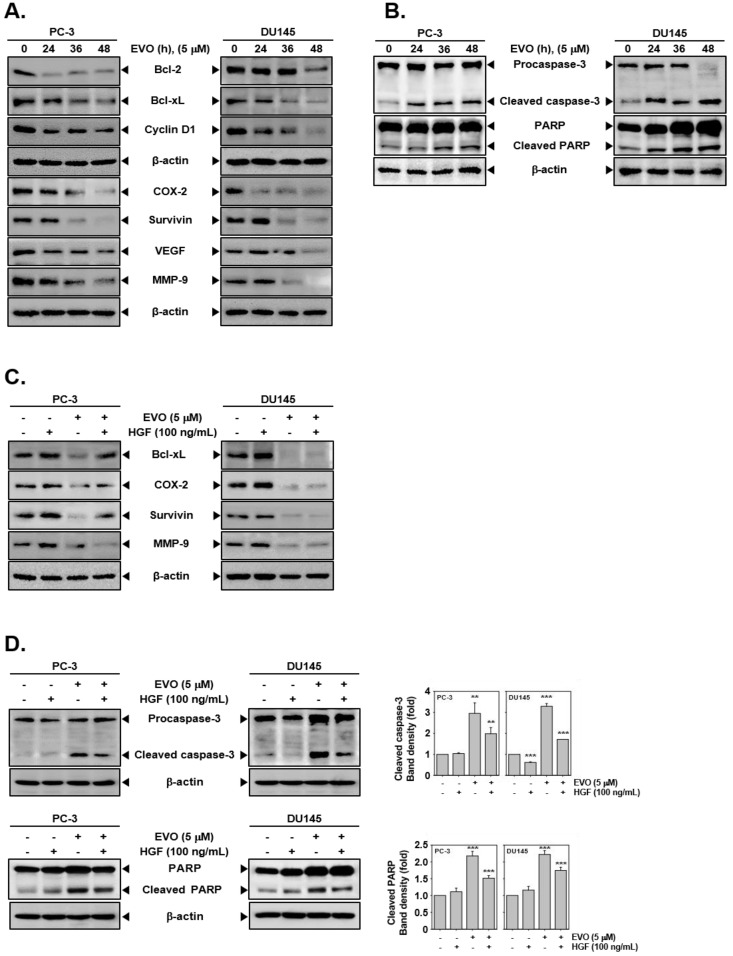
EVO suppresses the expression of various oncogenic proteins involved in survival, proliferation, cell cycle, angiogenesis, and metastasis and induces caspase-3 activation leading to the appearance of cleaved poly(ADP-ribose) polymerase (PARP)**.** (**A**) PC-3 (5 × 10^5^ cells/well) and DU145 cells (5 × 10^5^ cells/well) were treated with 5 µM EVO for the indicated times. Whole cell extracts were prepared and immunoblotted with antibodies for B-cell lymphoma 2 (Bcl-2), B-cell lymphoma-extra large (Bcl-xL), cyclin D1, cyclooxygenase 2 (COX-2), survivin, vascular endothelial growth factor (VEGF), and matrix metallopeptidase 9 (MMP-9). (**B**) PC-3 (5 × 10^5^ cells/well) and DU145 cells (5 × 10^5^ cells/well) were treated with 5 µM EVO for the indicated times. Whole cell extracts were prepared and immunoblotted with antibodies for cleaved caspase-3, caspase-3, cleaved PARP, and PARP. (**C**) PC-3 (5 × 10^5^ cells/well) and DU145 cells (5 × 10^5^ cells/well) were pre-treated with EVO (5 µM) for 1 h then treated with HGF (100 ng/mL) or left untreated for 36 h. Whole cell extracts were prepared and immunoblotted with antibodies for Bcl-xL, COX-2, survivin, and MMP-9. (**D**) PC-3 (5 × 10^5^ cells/well) and DU145 cells (5 × 10^5^ cells/well) were pre-treated with EVO (5 µM) for 1 h then treated with HGF (100 ng/mL) or left untreated for 36 h. Whole cell extracts were prepared and immunoblotted with antibodies for cleaved caspase-3, caspase-3, cleaved PARP, and PARP. NT and EVO (−)/HGF (−) indicate control samples treated with medium containing 0.1% DMSO.

As shown in Figure 3A, EVO substantially downmodulated the expression of various proteins involved in cell survival (B-cell lymphoma 2 (Bcl-2), B-cell lymphoma-extra large Bcl-xL, survivin), proliferation (cyclooxygenase 2 (COX-2)), cell cycle (cyclin D1), angiogenesis (vascular endothelial growth factor (VEGF)), and metastasis (matrix metallopeptidase 9 (MMP-9)) in a time-dependent manner. In addition, EVO effectively activated caspase-3 and led to poly(ADP-ribose) polymerase (PARP) breakdown in a time-dependent manner (Figure 3B). Interestingly, EVO not only reduced HGF-induced expression of anti-oncogenic proteins but also enhanced the levels of pro-apoptotic proteins (Figure 3C,D).

### 3.5. c-Met Knockdown Blocked Src/STAT3 Signaling in Prostate Cancer Cells.

**Figure 4:**
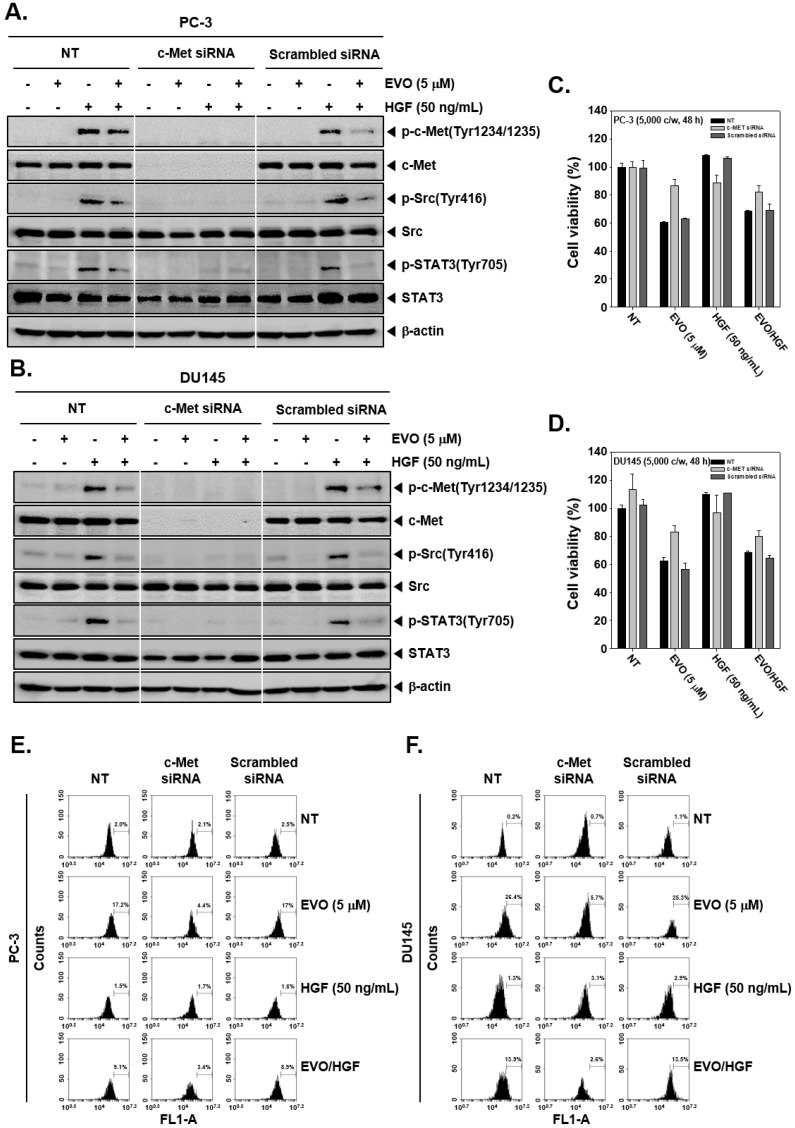
Silencing of c-Met in PC-3 and DU145 cells though c-Met small interfering RNA (siRNA) transfection. (**A** and **B**) PC-3 and DU145 cells were transfected with c-Met and scramble siRNA (100 nM) for 24 h using electroporation. The transfected cells were pre-treated with EVO (5 µM) for 4 h and then treated with HGF (50 ng/mL) for 15 min. Then, equal amounts of whole cell lysate were analyzed by Western blot using antibodies against phospho-c-Met(Tyr1234/1235) and c-Met. After overnight incubation in serum-free conditions following transfection, the cells were pre-treated with EVO (5 µM) for 4 h and then treated with HGF (50 ng/mL) for 15 min in serum-free conditions. Then, equal amounts of whole cell lysate were analyzed by Western blot using antibodies against p-Src and p-STAT3. (**C** and **D**) Transfected PC-3 and DU145 cells were re-seeded in 96-well plates (5 × 10^3^ cells/well) and pre-treated with EVO (5 µM) for 1 h, then treated with HGF (50 ng/mL) or left untreated for 48 h. Cell viability was compared after the different treatments using the MTT assay. (**E** and **F**) Transfected cells were pre-treated with EVO (5 µM) for 1 h then treated with HGF (50 ng/mL) or left untreated for 36 h. The cells were fixed, stained with TUNEL assay reagent, and then analyzed by flow cytometry. NT and EVO (−)/HGF (−) indicate control samples treated with medium containing 0.1% DMSO.

We next elucidated if EVO can inhibit Src/STAT3 signal through blockage of c-Met signaling in prostate cancer cells. As shown in Figure 4A and B, c-Met siRNA-transfected cells did not express c-Met, while non-treated and scramble siRNA-transfected cells displayed c-Met expression after HGF stimulation. Furthermore, HGF-induced phosphorylation of Src and STAT3 also had no obvious effect on c-Met siRNA-transfected cells. These results suggest that Src/STAT3 signals are acting downstream of c-Met, and EVO effectively downregulates the phosphorylation of the c-Met/Src/STAT3 signaling axis.

### 3.6. c-MET Knockdown Increased Cell Viability and Decreased EVO-Induced Cell Death.

When then compared the viability of c-Met-transfected and control cells. We found that c-Met-transfected cells exhibited higher cell viability than the control groups (Figure 4C and D). In addition, transfected PC-3 and DU145 cells were pre-treated with EVO (5 µM) for 1 h and then stimulated with HGF (50 ng/mL) or left untreated for 36 h. As shown in Figure 4E and F, c-Met siRNA-transfected cells showed a lower percentage of apoptotic cell death upon EVO treatment in comparison with control and scramble siRNA-transfected cells, as measured by TUNEL assay.

## 4. Discussion

**Figure 5:**
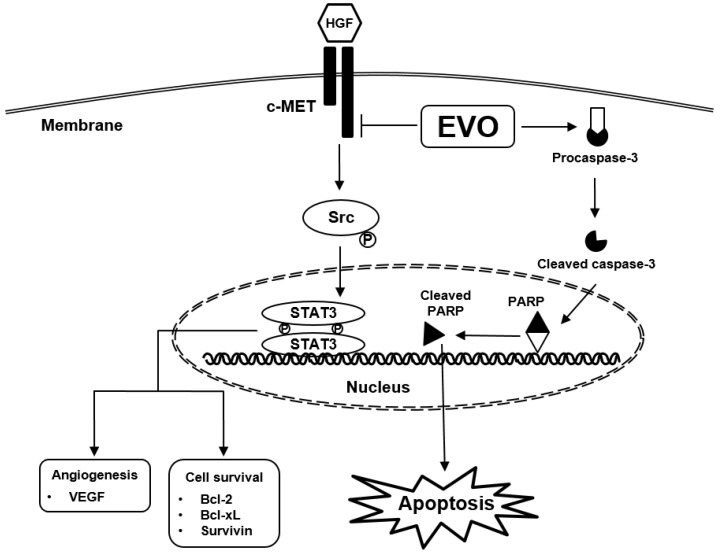
EVO downregulates HGF-induced c-Met/Src/STAT3 phosphorylation and causes apoptosis through blocking the c-Met signaling pathway.

The aim of this report was to examine the anti-cancer activity of EVO in androgen-independent prostate cancer. In previous studies, EVO displayed diverse pharmacological actions in various cancer cells including prostate cancer cells [38,51,52,53,54], but the detailed mechanism of its action of remains obscure. Thus, we investigated if EVO can exhibit cytotoxicity against tumor cells and whether its anti-neoplastic actions can be mediated by the targeted abrogation of c-Met-dependent signal cascade (Figure 5).

Activation of HGF/c-Met signal can regulate prostate cancer progression and maintain cellular growth [11,55,56]. We first examined the effective concentration of EVO using a cell viability assay and we determined the range of concentration (1, 2.5, and 5 µM) to be used in different assays. EVO caused higher cytotoxicity and exerted greater anti-proliferation and anti-cell growth actions in DU145 cells compared to PC-3 cells. Next, we investigated the effect of EVO on HGF-induced c-Met phosphorylation. EVO inhibited not only c-MET phosphorylation but also HGF-induced Src/STAT3 signaling, that is deregulated in various malignancies [57,58,59,60,61,62,63]. EVO substantially suppressed HGF-induced c-Met/Src/STAT3 phosphorylation and the attenuated the nuclear translocation of STAT3 protein. Additionally, we investigated that EVO effect was not consistent in other mechanisms. As shown in Figure S1, that only Akt signal decreased in PC-3 cells and Akt and MAPKs signal decreased in DU145 cells. Therefore, other mechanisms (MAPKs, PI3K/Akt, and NF-κB) were not mainly signaling pathway and we focused on Src/STAT3 signaling in our study.

We found that EVO can also effectively enhance apoptosis, by performing cell cycle analysis and TUNEL assay. These results may be different from those of prior studies in which EVO was reported to cause cell cycle arrest in G2/M phase [14,54,64], while, according to our cell cycle analysis results, EVO treatment led to sub-G1 phase accumulation of tumor cells in a time-dependent manner. In addition, we observed that in HGF-co-treated cells, there was a decrease of cells in sub-G1 phase, which led to the confirmation that EVO regulates the cell cycle by inhibiting c-Met signaling. Moreover, we also found that HGF-co-treated cells displayed decreased apoptosis than cells treated with EVO alone, as shown by the results of the TUNEL assays performed in the same conditions as cell cycle analysis. These TUNEL assay results are consistent with those of other previous studies [14,54].

EVO also reduced the expression of c-Mer/Src/STAT3-regulated carcinogenic molecules, including those inhibiting apoptosis and regulating proliferation, angiogenesis, and metastasis in PC-3 and DU145 cells. Theses oncogenic proteins can protect tumor cells from apoptosis [6,65] and mediate malignant transformation [66]. Furthermore, we found that EVO suppressed c-Met/Src/STAT3-regulated oncogenic proteins even in the presence of HGF. Further, the activation of caspase-3 and PARP cleavage were observed in the presence or absence of HGF upon EVO exposure.

Besides, to analyze if EVO can inhibit the Src/STAT3 signaling axis by interrupting c-Met signaling, transfection experiments were carried out. We observed that in c-Met siRNA-transfected cells, the effect of EVO on HGF-induced c-Met/Src/STAT3 phosphorylation was neutralized. In addition, the percentage of apoptotic cells decreased and cell viability increased in c-Met siRNA-transfected cells with respect to control cells. Overall, EVO can attenuate the activation of c-Met/Src/STAT3 pathways and act as a modulator of cancer cell survival, proliferation, and angiogenesis.

## Supplementary Materials

The following are available online at https://www.mdpi.com/1420-3049/25/6/1320/s1, Figure S1: Effect of EVO in others signaling cascades such as MAPKs, PI3K/Akt, and NF-κB. (A–B) Both cells were seeded in six-well plates and incubated overnight in serum-free conditions. Then, they were pre-treated with EVO for 4 h and treated with HGF for 15 min in serum-free conditions. Whole cell extracts were prepared and immunoblotted with indicated antibodies. (C) Both cells were prepared same condition as previous. Nuclear extracts and cytoplasmic extracts were prepared and immunoblotted with indicated antibodies.

## Abbreviations

HGF:	hepatocyte growth factor
PARP:	Poly(ADP-ribose) polymerase
Bcl-2:	B-cell lymphoma 2
VEGF:	Vascular endothelial growth factor
COX-2:	Cyclooxygenase 2
MMP-9:	Matrix metallopeptidases 9
GAPDH:	Glyceraldehyde 3-phosphate dehydrogenase
TUNEL:	TdT-mediated dUTP nick-end labeling
SDS:	Sodium dodecyl sulfate
PBS:	Phosphate buffered saline
TBS:	Tris buffered saline
ECL:	Enhanced chemiluminescence
PI:	Propidium iodide
FBS:	Fetal bovine serum
MTT:	3-(4,5-dimethylthiazol-2-yl)-2,5-diphenyltetrazolium bromide
siRNA:	Small interfering RNA
