# Supplementary material for: Evodiamine Mitigates Cellular Growth and Promotes Apoptosis by Targeting the c-Met Pathway in Prostate Cancer Cells
Source: Molecules. 2020 Mar 13;25(6):1320. doi: 10.3390/molecules25061320 (PMC7144730; doi:10.3390/molecules25061320)
Supplement: Supplementary file 1 [file molecules-25-01320-s001.pdf]

Supplementary Materials

Article

Evodiamine mitigates cellular growth and promotes apoptosis by targeting the c-Met pathway in prostate cancer cells

Sun Tae Hwang [1], Jae-Young Um [1], Arunachalam Chinnathambi [2], Sulaiman Ali Alharbi [2], Acharan S. Narula [3], Ojas A. Namjoshi [4], Bruce E. Blough [4] and Kwang Seok Ahn [1,*]

[1] Department of Science in Korean Medicine, Kyung Hee University, 24 Kyungheedae-ro, Dongdaemun-gu, Seoul 02447, Republic of Korea; suntaeh12@gmail.com (S.T.H.), jyum@khu.ac.kr (J.-Y.U.)

[2] Department of Botany and Microbiology, College of Science, King Saud University, Riyadh -11451, Kingdom of Saudi Arabia; carunachalam@ksu.edu.sa (A.C.), sharbi@ksu.edu.sa (S.A.A.)

[3] Narula Research, Chapel Hill, NC 27516, USA, anarula1@nc.rr.com

[4] Center for Drug Discovery, RTI International, Research Triangle Park, Durham, NC, USA 27616, onamjoshi@rti.org (O.A.N.), beb@rti.org (B.E.B.)

* Correspondence: ksahn@khu.ac.kr Phone: 82-2-961-2316

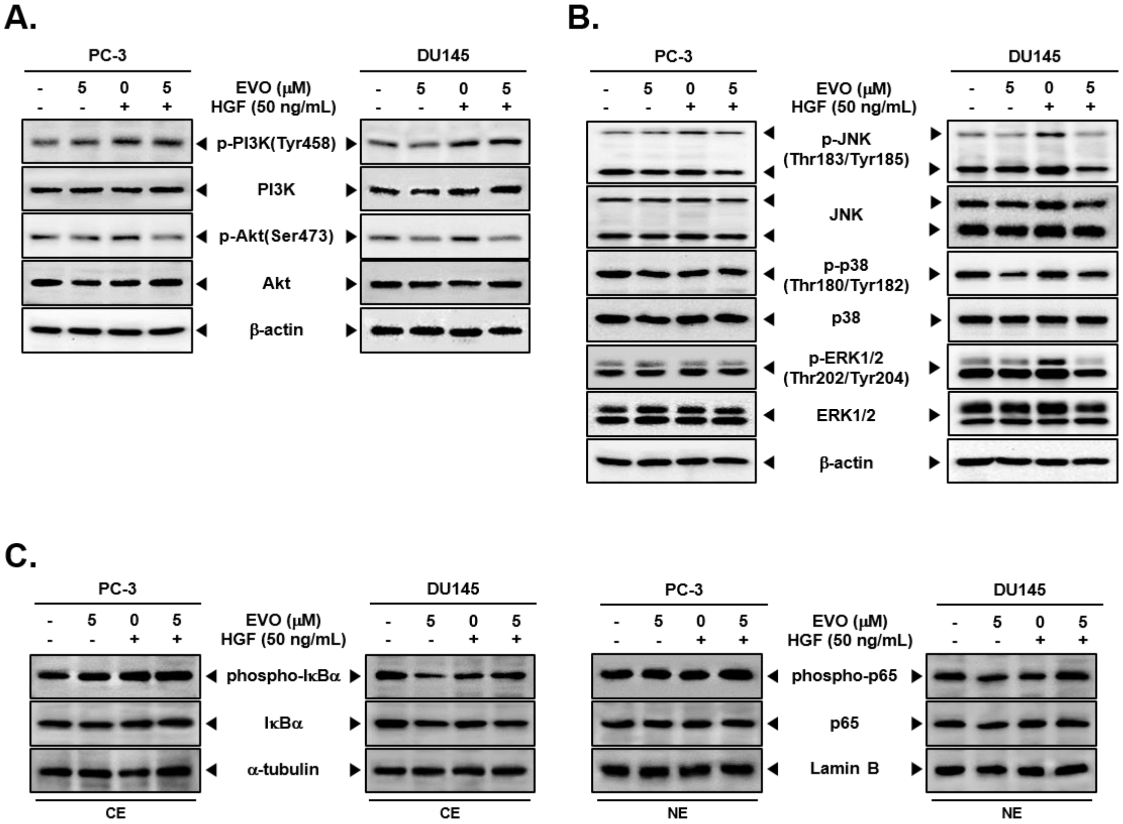

Figure S1. Effect of EVO in others signaling cascades such as MAPKs, PI3K/Akt, and NF-κB. (**A**–**B**) Both cells were seeded in six-well plates and incubated overnight in serum-free conditions. Then, they were pre-treated with EVO for 4 h and treated with HGF for 15 min in serum-free conditions. Whole cell extracts were prepared and immunoblotted with indicated antibodies. (**C**) Both cells were prepared same condition as previous. Nuclear extracts and cytoplasmic extracts were prepared and immunoblotted with indicated antibodies.
